# Research on sentiment classification for netizens based on the BERT-BiLSTM-TextCNN model

**DOI:** 10.7717/peerj-cs.1005

**Published:** 2022-06-08

**Authors:** Xuchu Jiang, Chao Song, Yucheng Xu, Ying Li, Yili Peng

**Affiliations:** 1 Zhongnan University of Economics and Law, Wuhan, Hubei, China; 2 Wuhan Institute of Technology, Wuhan, Hubei, China

**Keywords:** BERT, BiLSTM, TextCNN, Sentiment classification

## Abstract

Sentiment analysis of netizens’ comments can accurately grasp the psychology of netizens and reduce the risks brought by online public opinion. However, there is currently no effective method to solve the problems of short text, open word range, and sometimes reversed word order in comments. To better solve the above problems, this article proposes a hybrid model of sentiment classification, which is based on bidirectional encoder representations from transformers (BERT), bidirectional long short-term memory (BiLSTM) and a text convolution neural network (TextCNN) (BERT-BiLSTM-TextCNN). The experimental results show that (1) the hybrid model proposed in this article can better combine the advantages of BiLSTM and TextCNN; it not only captures local correlation while retaining context information but also has high accuracy and stability. (2) The BERT-BiLSTM-TextCNN model can extract important emotional information more flexibly in text and achieve multiclass classification tasks of emotions more accurately. The innovations of this study are as follows: (1) the use of BERT to generate word vectors has the advantages of more prior information and a full combination of contextual semantics; (2) the BiLSTM model, as a bidirectional context mechanism model, can obtain contextual information well; and (3) the TextCNN model can obtain important features well in the problem of text classification, and the combined effect of the three modules can significantly improve the accuracy of emotional multilabel classification.

## Introduction

With the development of big data, cloud computing and 5G technology, social media pay more attention to data mining, sorting and utilization to provide users with accurate personalized services. More netizens can express their ideas and spread their ideas and opinions on microblog and other platforms. At this stage, it is important to obtain emotional analysis results through natural language processing. Recurrent neural networks can make full use of contextual information and preserve the sequence of sentences to achieve sentiment classification and polysemy; pretraining models save subsequent development time and difficulty in use and are very useful in the recent natural language processing (NLP) field, but they require considerable data support, and the algorithm training time is very long. With the development of big data and artificial intelligence and the emergence of various emerging media, there is a large amount of data to support the use of deep learning, and an increasing number of new words will appear, which can accurately judge the meaning of words and play a crucial role in analysis and decision-making (Cambria, 2016). The key problems faced in the research of NLP sentiment analysis are as follows. In this context, the existence of irony has not yet found a better way to deal with it; most of the classifications still use two-category sentiment analysis. Analysis has not worked well. Through the analysis of existing research, this article proposes the bidirectional encoder representations from transformers (BERT), bidirectional long short-term memory (BiLSTM) and a text convolution neural network (TextCNN) (BERT-BiLSTM-TextCNN) hybrid model. The experimental results show that the classification accuracy can be significantly improved, and the problem of semantic inversion has also been solved to a certain extent.

### Single neural network

In some previous studies, convolutional neural networks (CNNs) achieved good results, but CNNs do not consider the potential theme of the text. In 2014, Kim (2014) proposed applying a CNN model to the task of text classification and found that the TextCNN model can extract the semantic information of the text and capture the relevant information of the context. TextCNN has the features of simple structure, fast training speed and good effect. It is widely used in NLP fields such as text classification and recommendation. The Text Recurrent Neural Network (TextRNN) was proposed by Liu, Qiu & Huang (2016); it can capture the temporal characteristics of text and has a good effect on text classification tasks when compared with TextCNN, but its training speed is relatively slow. Cao et al. (2019) proposed a sentiment analysis method for Chinese text based on bidirectional gated recurrent units (BiGRUs). The text was first converted into a word vector sequence, and BiGRU was used for the contextual sentiment features of the text. The F1 value reached 90.61%, which was higher than that of the CNN method in terms of accuracy and training speed. To improve the training speed and reduce the cost, Wang et al. (2016) proposed a memory network classification based on attention for long-term and short-term aspect-oriented sentiments and classified sentiments based on long short-term memory (LSTM) networks. Gopalakrishnan & Salem (2020) proposed six simplified LSTM models with different parameters, compared the performance differences of these LSTM models and established the best parameter set for LSTM models. Chen et al. (2018) proposed a new emotional analysis scheme based on Twitter and Sina micro-blog data that considered the factors of sentiment caused by expressions. By taking part in these ambiguous expressions, an emotional classifier is trained and embedded into the attention-based long-term memory network, which has a good guiding effect on emotional analysis.

### Hybrid neural network

In addition to the study of a single neural network, many scholars combined the advantages of different neural networks and applied them to emotional analysis. Basiri et al. (2021) proposed an attention-based bidirectional CNN-RNN deep model (ABCDM). ABCDM uses two independent BiLSTM and GRU layers to extract the past and future contexts by considering the time information flow in two directions. At the same time, an attention mechanism is applied in the output of the ABCDM bidirectional layer to emphasize different words. Rehman et al. (2019) proposed a hybrid model using LSTM and depth CNN models. First, the Word2Vec method was used to train the initial word embedding, and then the feature set extracted by convolution was combined with the global maximum pool layer with long-term dependence to embed the postscript. The model also adopted dropout technology, normalization and correction linear units to improve the accuracy. Zhang et al. (2019) proposed a short text sentiment classification method based on a CNN-LSTM model that combines CNN with LSTM and compared it with the embedding long short-term memory (E-LSTM) method, which used 3D convolution instead of 2D convolution and then introduced BiE-LSTM (bidirectional embedding long short-term memory). Minaee, Azimi & Abdolrashidi (2019) proposed a sentiment analysis method based on CNN and BiLSTM models: one was used to capture the time information of data, the other was used to extract the local structure of data, and the effect was better than using the CNN-LSTM model alone. Li et al. (2021) proposed a hierarchical attention BiLSTM model based on cognitive brain (ALCB) for multimodal sentiment analysis. Finn, Abbeel & Levine (2017) proposed a sentiment classification method based on MAML (Model Agnostic Meta Learning) and BiLSTM, using gradient descent to update parameters. Compared with the current popular models, the accuracy, recall and F1 value of the aspect sentiment data set were increased by 1.68%, 2.86% and 2.27%, respectively. Gan, Feng & Zhang (2021) proposed a scalable multichannel dilated joint architecture of a CNN-BiLSTM model with an attention mechanism to analyze the sentiment tendency of Chinese texts. This method not only solved the problem of gradient disappearance or gradient explosion in the traditional RNN model but also made up for the problem that CNN could not effectively extract contextual semantic information and introduced an attention mechanism to better extract the local features of sentences. Cambria et al. (2022) proposed an AI framework which can make explainable sentiment analysis. The framework provides a new research inspiration for sentiment analysis.

The amount of information contained in the comment set is limited, but the content expressed is very rich. Both the important local features and contextual information of the text affect the category to which the sentiment belongs. In addition, a better representation of the word vector can greatly improve the classification accuracy of the model. Based on the above theories, the deep learning-based text classification method does not require manual feature engineering and can be trained with a larger data set so that more knowledge can be learned. This article is inspired by the bidirectional encoder representations from transformers (BERT), bidirectional long short-term memory (BiLSTM) and a conditional random fields (CRF) (BERT-BiLSTM-CRF) model proposed by Meng et al. (2022) and adds the TextCNN module on this basis. The BERT model is trained based on a large *corpus*. It can fully consider the semantic relationship when generating word vectors, and the generated word vectors have a dimension large enough to fully represent the meaning of words. The BiLSTM model adopts a bidirectional mechanism, which can fully consider the contextual features of the text and can learn the contextual information of important features in this article. The TextCNN model can obtain the local features of the text through convolution and add the maximum pooling layer to obtain important features in the text. Combining these advantages and disadvantages, this article uses a fusion of BERT, BiLSTM, and TextCNN models and makes full use of various information in the reviews, including contextual features and word vector content, to classify the sentiment of the review text through deep learning.

## Model

### Research framework

**Figure 1:**
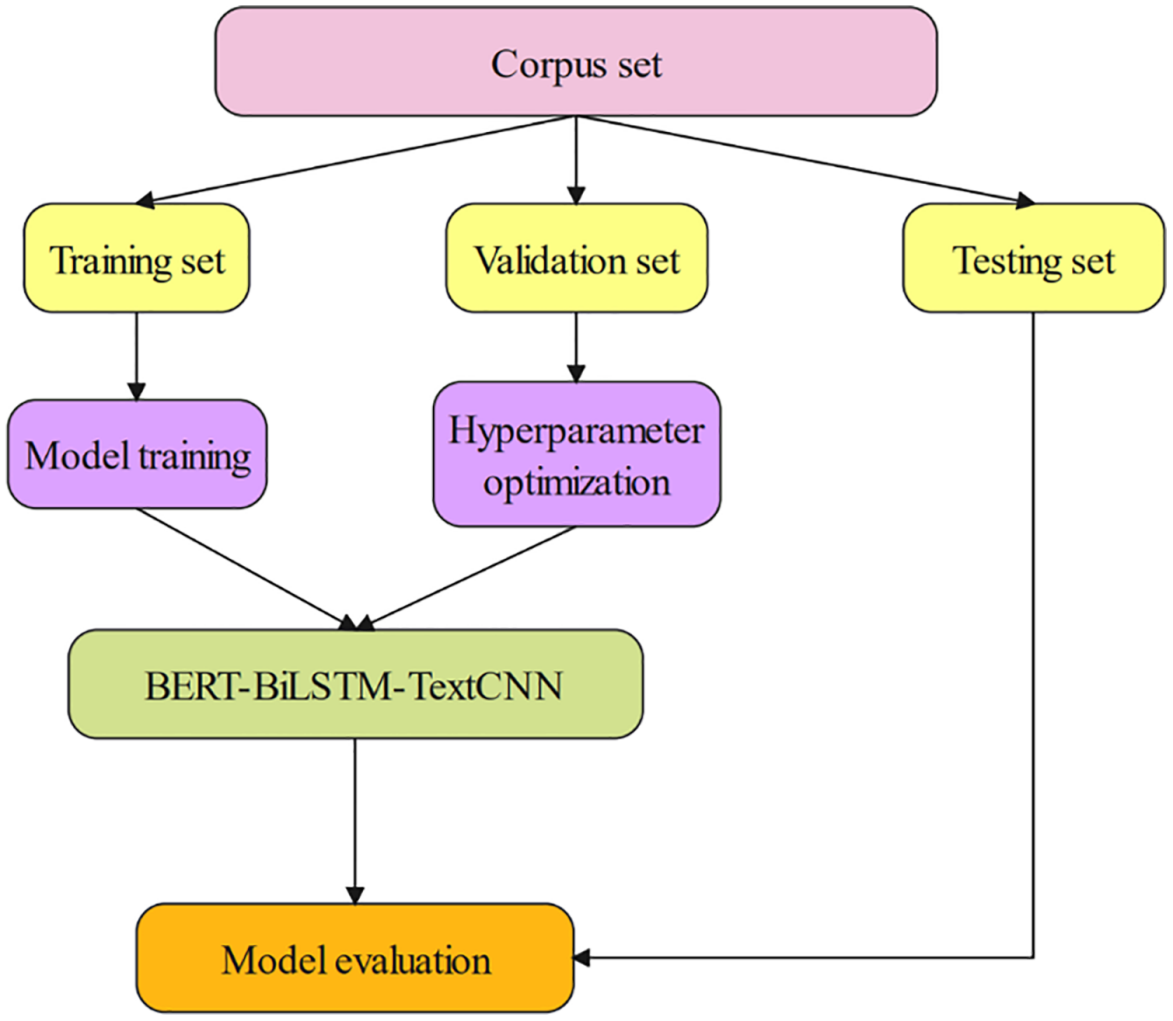
Research framework.

Figure 1 shows the research framework of netizen sentiment classification based on the BERT-BiLSTM-TextCNN model. First, divide the data set into a training set and a validation set. Then, the built model is trained, and the validation set is used for hyperparameter selection. Finally, the test set is used to evaluate the model according to the two indicators of Micro-F1 and Macro-F1. To highlight the effectiveness and accuracy of the model, seven models were selected for comparison, including BERT-BiGRU-TextCNN, BERT-LSTM-TextCNN, and Word2Vec-BiLSTM-TextCNN.

### Model building

**Figure 2:**
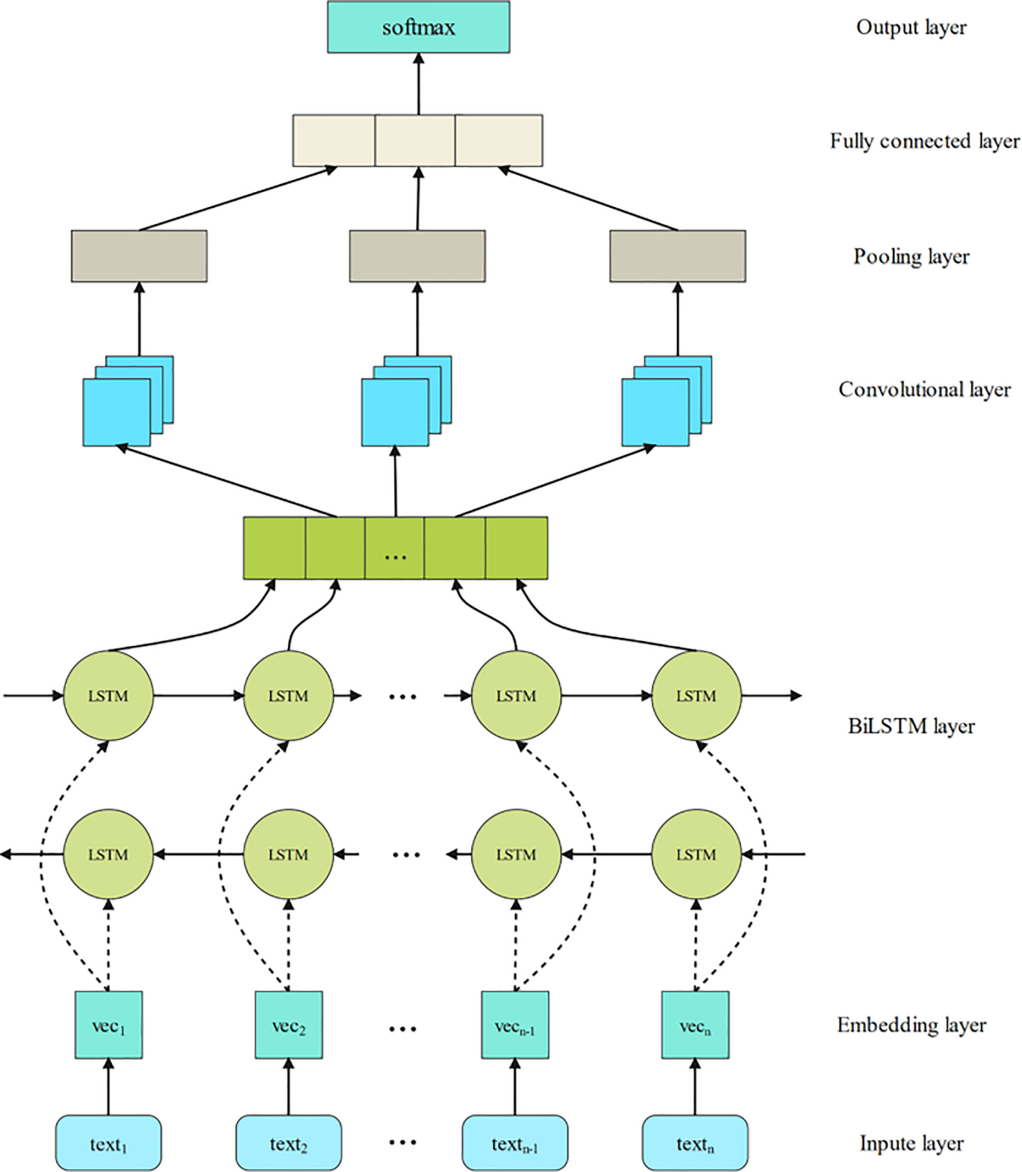
BERT-BiLSTM-TextCNN model.

Building a neural network is the key to this experiment. The specific model structure is shown in Fig. 2. When performing sentiment analysis, natural language is first digitized to facilitate subsequent processing. Since different mapping methods will have a huge impact on the result, how to choose the word embedding method and map the natural language into a better word vector is a crucial step. BERT uses two-way pretraining, which solves the limitation of the following information in the prediction of the generative pretraining (GPT) model to prevent leakage. Therefore, this article selects BERT in the word embedding layer to convert the text into a word vector.

Since BiLSTM can capture text information from both the forward direction and the reverse direction at the same time, TextCNN uses multiple convolution kernels of different sizes to extract key information in sentences (similar to n-grams with multiple window sizes), which can better capture local correlation, so this article combines the two models. Input the obtained word vector into the BiLSTM layer for bidirectional learning, input the obtained result into the convolutional layer, and perform feature extraction. After that, they pass through the pooling layer while maintaining the main features, greatly reducing the number of parameters and overfitting. Finally, the results are spliced and put into the fully connected layer for output, and the sentiment analysis result is obtained. Specific modules can be divided into six parts.

1. Remove punctuation or special symbols from the collected data, convert it into a specific format, and then input it into the BERT word embedding layer. In this layer, each piece of data is processed into data with a sentence dimension of 64 and a word vector dimension of 768. These data are then fed into the BiLSTM layer.
2. The data are input to the BiLSTM layer to extract the contextual features of the text. This layer is activated using the rectified linear units (ReLU) function. The LSTM output vector dimension in the front and rear directions is 128. Since it is bidirectional LSTM splicing, the output vector dimension of this layer is 256.
3. After the context feature extraction, input to the TextCNN layer to further extract the important features of the text. The window widths of this layer are 3, 4, and 5. The ReLU function is used for activation, and the parameter (padding = same) is used to avoid the phenomenon of different dimensions of the output vector due to different window sizes during convolution and to ensure the three outputs. The dimensions are the same as the input dimensions, which is convenient for subsequent operations. Using three different window widths can learn more local feature information.
4. The vector output by TextCNN is input to the maximum pooling layer. The window size of the maximum pooling layer is two, and the step size is two. This setting makes the final sentence vector dimension only halved, and the context relationship can be preserved to a certain extent.
5. The outputs of TextCNN with three window sizes after max-pooling at the fully connected layer are concatenated together.
6. The last layer is the softmax text classification layer. This layer receives the data from the fully connected layer and classifies it, and the dimensions of each vector obtained are four dimensions. The category of emotion can be determined by comparing the magnitude of each value of the output four-dimensional vector.

#### Embedding layer on BERT

In BERT, the embedding layer has three forms, including token embedding, segment embedding and position embedding. The input text is first tokenized and then represented as a 768-dimensional vector in the token embedding layer. In addition, extra tokens are added at the beginning ([CLS]) and ending ([SEP]) of the tokenized phrase to represent the tasks of classification and isolating pairs of input texts. Segment embedding is used to distinguish two similar texts that have only two kinds of vectors. The former vector assigns zero to each token in the first sentence, and the latter vector assigns one to each token in the second sentence. If the input text only has a single sentence, then its segment embedding is zero. The last layer is positive embedding, which is used to describe the position of each word in the sentence. The above three vector representations are added tokenwise to obtain a composite representation of size (1, n, 768), which is the final input to the BERT embedding layer.

#### Bidirectional long short-term memory layer

In this layer, it will be used to process the vectors obtained by the pretrained BERT model for forward and reverse learning. The BiLSTM model replaces the traditional hidden layer with the LSTM layer, which can obtain two kinds of information of the cell state and the hidden layer state from the previous moment and adopts the control gate mechanism. It is composed of the memory cell ${i_t}$, the input gate ${C_t}$, the output gate ${O_t}$, and the forgetting gate ${f_t}$. The calculation process of LSTM can be summarized as follows: cross the forgetting gate ${f_t}$ and memory gate ${i_t}$ in the cell state, filter and transmit useful information for subsequent calculations, discard useless information, and output the hidden layer state ${h_t}$ at each time step.

The specific definition is shown in Eqs. (1)–(6)

(1) $${f_t} = {\rm sigmoid}{({{\rm w}_f}{{[h_{t - 1}, {x_t]} + {b_f})}}}$$

(2) $${i_t} = {\rm sigmoid}{({{\rm w}_i}{{[h_{t - 1}, {x_t]} + {b_i})}}}$$

(3) $$\widetilde {{C_t}} = {\rm tanh}{({\rm w}_c[{h_{t-1}},\,{x_t}]+{b_c})}$$

(4) $${C_t} = {f_t} \times {C_{t - 1}} + {i_t} \times \widetilde {{C_t}}$$

(5) $${O_t}={\rm sigmoid}{({\rm w}_o{[h_{t-1},\,{x_t}]+{b_o}})}$$

(6) $${h_t} = {O_t} \times {\rm tanh}\left( {{C_t}} \right)$$

The key to solving the gradient dispersion problem is to forget some unimportant information and retain key information.

#### Text convolutional neural network model

In the convolution layer, this layer uses convolution kernels of different sizes. In a matrix with size $n \times h$, for a given convolution kernel ${\rm \omega } \in {R^{hk}}$ and a window ${x_{i:i + h - 1}}$, the convolution operation is performed to generate a feature function ${c_i} = f\left( {{\rm \omega } \cdot {x_{i:i + h - 1}} + b} \right)$. In this function, ${x_{i:i + h - 1}}$ represents a window of size $h \times k$ composed of rows $i$ to $i + h - 1$ of the input matrix, which is spliced by ${x_i},{x_{i + 1}}, \ldots ,{x_{i + h - 1}}$, $h{\rm \; }$represents the number of words in the window, ${\rm \omega }$ is the weight matrix, *b* is the bias parameter, and $f$ is the nonlinear function. Each convolution operation means a feature vector extraction. By defining different windows, different feature vectors can be extracted to form the output of the convolution layer. This article selects the 1-Max pooling method in the pooling layer and a maximum feature from the feature vector generated by each sliding window and then concatenates it to form a vector representation. The final fully connected layer, which uses the softmax function for classification, is shown in Eq. (7).

(7) $${P_i} = \displaystyle{{{e^i}} \over {\mathop \sum \nolimits^j {e^i}}}$$

where ${P_i}$ denotes the probability of the ${i_{th}}$ class, ${e^i}$ indicates the corresponding value of the output of the ${i_{th}}$ class and $j$ denotes the total number of classes.

## Experimental Analysis

### Data sources

There are two sources of data in this article. One of them comes from GitHub’s public data set “simplyweibo_4_moods” (https://github.com/SophonPlus/ChineseNlpCorpus/tree/master/datasets/simplifyweibo_4_moods), which contains more than 360,000 comments of Sina micro-blog with sentiment labels, including four sentiments: happy (200,000 comments), angry (50,000 comments), disgusted (50,000 comments) and depressed (50,000 comments).

In addition, by crawling micro-blog comments related to the Longping Yuan incident, the collection time was set from 21 May 2021, to 27 May 2021, and 240,000 comments related to Longping Yuan’s death were crawled. By cleaning the text and removing special characters, the comment corpora were obtained. According to the theory of public opinion communication and micro-blog’s intelligent recommendation algorithm, the incident can be divided into three stages: (1) the outbreak peak period: from 21 May 2021 to 22 May 2021; (2) the comprehensive spreading period: from 23 May 2021 to 24 May 2021; and (3) the control recovery period: from 25 May 2021 to 27 May 2021.

### Evaluation criteria

To quantitatively evaluate the prediction performance of the models, this article selects the Precision, Recall, F1, Micro-F1 and Macro-F1 values to measure the prediction accuracy and generalization ability of different models, assuming TP as true positive, FP as false positive, TN as true negative and FN as false negative. *i* is sentiment category (*i* = 0, 1, 2, 3). This leads to the definition formula.

(8) $${\rm Precision} = \displaystyle{{{\rm TP}} \over {{\rm TP} + {\rm FP}}}$$

(9) $${\rm Recall} = \displaystyle{{{\rm TP}} \over {{\rm TP} + {\rm FN}}}$$

(10) $${\rm F} = 2 \cdot \displaystyle{{{\rm Precision} \cdot {\rm Recall}} \over {{\rm Precision} + {\rm Recall}}}$$

However, for the multiclass classification problem, it is inaccurate to rely on the above indicators only, so it is necessary to introduce Micro-F1 and Macro-F1. ${\rm Precisio}{{\rm n}_{{\rm micro}}} = \displaystyle{{\mathop \sum \nolimits_{i = 1}^n {\rm T}{{\rm P}_i}} \over {\mathop \sum \nolimits_{i = 1}^n {\rm T}{{\rm P}_i} + \mathop \sum \nolimits_{i = 1}^n {\rm F}{{\rm P}_i}}}$, ${\rm Recal}{{\rm l}_{{\rm micro}}} = \displaystyle{{\mathop \sum \nolimits_{i = 1}^n {\rm T}{{\rm P}_i}} \over {\mathop \sum \nolimits_{i = 1}^n {\rm T}{{\rm P}_i} + \mathop \sum \nolimits_{i = 1}^n {\rm F}{{\rm N}_i}}}$, ${\rm Precisio}{{\rm n}_{{\rm macro}}} = \displaystyle{1 \over n} \cdot \mathop \sum \limits_{i = 1}^n {\rm Precisio}{{\rm n}_i}$, ${\rm Recal}{{\rm l}_{{\rm macro}}} = \displaystyle{1 \over n} \cdot \mathop \sum \limits_{i = 1}^n {\rm Recal}{{\rm l}_i}$. The specific definition is shown in Eqs. (11) and (12).

(11) $${\rm Micro - F1} = 2 \cdot \displaystyle{{{\rm Precisio}{{\rm n}_{{\rm micro}}} \cdot {\rm Recal}{{\rm l}_{{\rm micro}}}} \over {{\rm Precisio}{{\rm n}_{{\rm micro}}} + {\rm Recal}{{\rm l}_{{\rm micro}}}}}$$

(12) $${\rm Macro - F1} = 2 \cdot \displaystyle{{{\rm Precisio}{{\rm n}_{{\rm macro}}} \cdot {\rm Recal}{{\rm l}_{{\rm macro}}}} \over {{\rm Precisio}{{\rm n}_{{\rm macro}}} + {\rm Recal}{{\rm l}_{{\rm macro}}}}}$$

### Experimental setup

The public data set “simplyweibo_4_moods” is selected from GitHub in this experiment. Each emotion is uniformly randomly sampled according to the ratio of 98:1:1 to divide into the training set, the validation set and the test set. The training set is used to train the model and determine the learning parameters of the model; the validation set is used to determine the hyperparameters to select the optimal model. The test set is used to evaluate the final model after training is completed. To obtain the best model effect, the hyperparameters of the model are adjusted. The specific results are shown in Table 1. When setting the parameters of the BiLSTM layer, the learning rate is set to 0.001, and the number of training rounds is set to 15. Due to the large number of texts, the batch size is set to 300, Adam is used as the optimization function to accelerate the convergence, and cross entropy is used as the loss function.

**Table 1:** Hyperparameter tuning.

BiLSTM layer		TextCNN layer	
Parameters	Values	Parameters	Values
Hidden node problem	300	Convolution kernels number	300
Learning rate	0.001	Convolution kernels size	3, 4, 5
Epochs	15	Activation function	ReLU
Batch_size	300	Pooling strategy	1-max pooling
Optimization function	Adam	Dropout	0.5
Loss function	Cross entropy	L2 regularization	Three
Input word vector	BERT		

At the TextCNN layer, due to the short review corpora, this article uses a smaller convolution kernel. After finding the best single convolution kernel, trying to find other suitable values around this value to combine results is better than a single best convolution kernel, so the combination of convolution kernels (3, 4, 5) is selected. The number of each convolution kernel is 100, and there are 300 convolution kernels in total. To reduce the risk of overfitting, dropout is set to 0.5. The activation function uses the ReLU function, the 1-max pooling strategy performs better, and the L2 regularization is set to 3.

**Figure 3:**
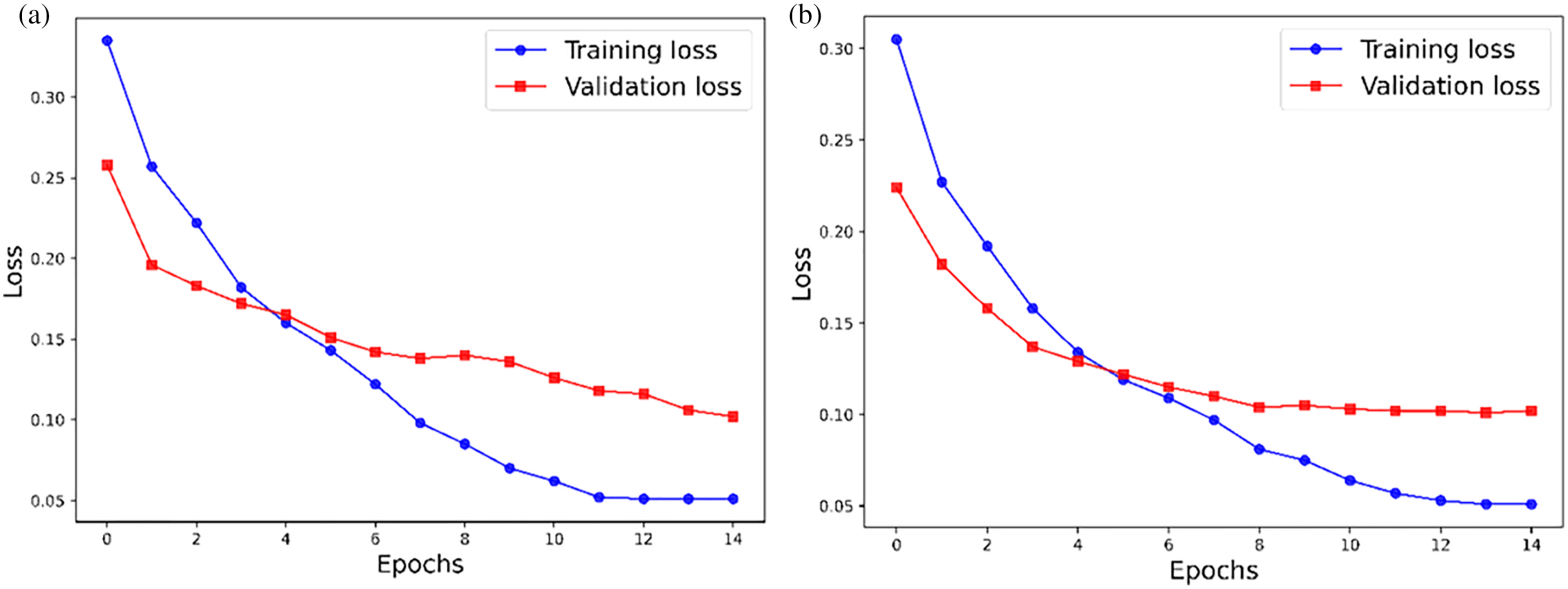
Comparison (A) before and (B) after tuning.

In this article, the training set and the validation set are characterized by loss before and after tuning, for a total of 15 rounds. The specific comparison is shown in Fig. 3. Figures 3A and 3B show the loss comparison of the training set and verification set before and after tuning. After tuning, the loss images of the training set and the validation set are smoother, converge faster, and the model performs better.

### Sentiment classification

The data set was imported into BERT-BiLSTM-TextCNN for training, the test set was applied to model evaluation, and the values of Precision, Recall, F1, Micro-F1 and Macro-F1 under each category were obtained. Compared with the other seven models, such as BERT-BiGRU-TextCNN, the results are shown in Table 2. Categories 0, 1, 2, and 3 represent happy, angry, detest, and depressed, respectively. Since Micro-F1 considers the number of various categories, a larger number of categories has a greater impact on it. Macro-F1 averages the Precision and Recall of each category and does not consider the amount of data. Therefore, the category with higher Precision and Recall will have a greater impact on Macro-F1. Since the number of texts in each category is consistent, Macro-F1 is higher than Micro-F1. All indicators of the target model BERT-BiLSTM-TextCNN are generally higher than those of the comparison model, which proves the effectiveness of the model.

**Table 2:** Model evaluation.

Model	Category	Precision	Recall	F1	Micro-F1	Macro-F1
BERT-BiLSTM-TextCNN	0	0.9235	0.9085	0.9159	0.9052	0.9143
	1	0.9146	0.9047	0.9096		
	2	0.9184	0.9130	0.9157		
	3	0.9296	0.9022	0.9157		
BERT-BiGRU-TextCNN	0	0.9105	0.9029	0.9067	0.8793	0.8885
	1	0.9062	0.8953	0.9007		
	2	0.8883	0.8784	0.8833		
	3	0.8542	0.8721	0.8631		
BERT-LSTM-TextCNN	0	0.8724	0.8951	0.8836	0.8629	0.8785
	1	0.8843	0.8765	0.8804		
	2	0.9023	0.8701	0.8859		
	3	0.8528	0.8742	0.8634		
BERT-TextCNN	0	0.8749	0.8412	0.8577	0.8598	0.8757
	1	0.8852	0.8685	0.8768		
	2	0.8537	0.8821	0.8677		
	3	0.9043	0.8957	0.9000		
Word2Vec-BiLSTM-TextCNN	0	0.7103	0.7348	0.7223	0.6300	0.6723
	1	0.6892	0.6438	0.6657		
	2	0.6719	0.7087	0.6898		
	3	0.6155	0.6043	0.6098		
Word2Vec-BiGRU-TextCNN	0	0.6361	0.6207	0.6283	0.6075	0.6265
	1	0.6345	0.6531	0.6437		
	2	0.6394	0.6112	0.6250		
	3	0.6145	0.6026	0.6085		
Word2Vec-LSTM-TextCNN	0	0.6581	0.6323	0.6449	0.6189	0.6231
	1	0.6361	0.6129	0.6243		
	2	0.6138	0.6199	0.6168		
	3	0.6037	0.6084	0.6060		
Word2Vec-TextCNN	0	0.6326	0.6109	0.6216	0.6010	0.6145
	1	0.6370	0.6211	0.6289		
	2	0.6024	0.6029	0.6026		
	3	0.6018	0.6075	0.6046		

**Figure 4:**
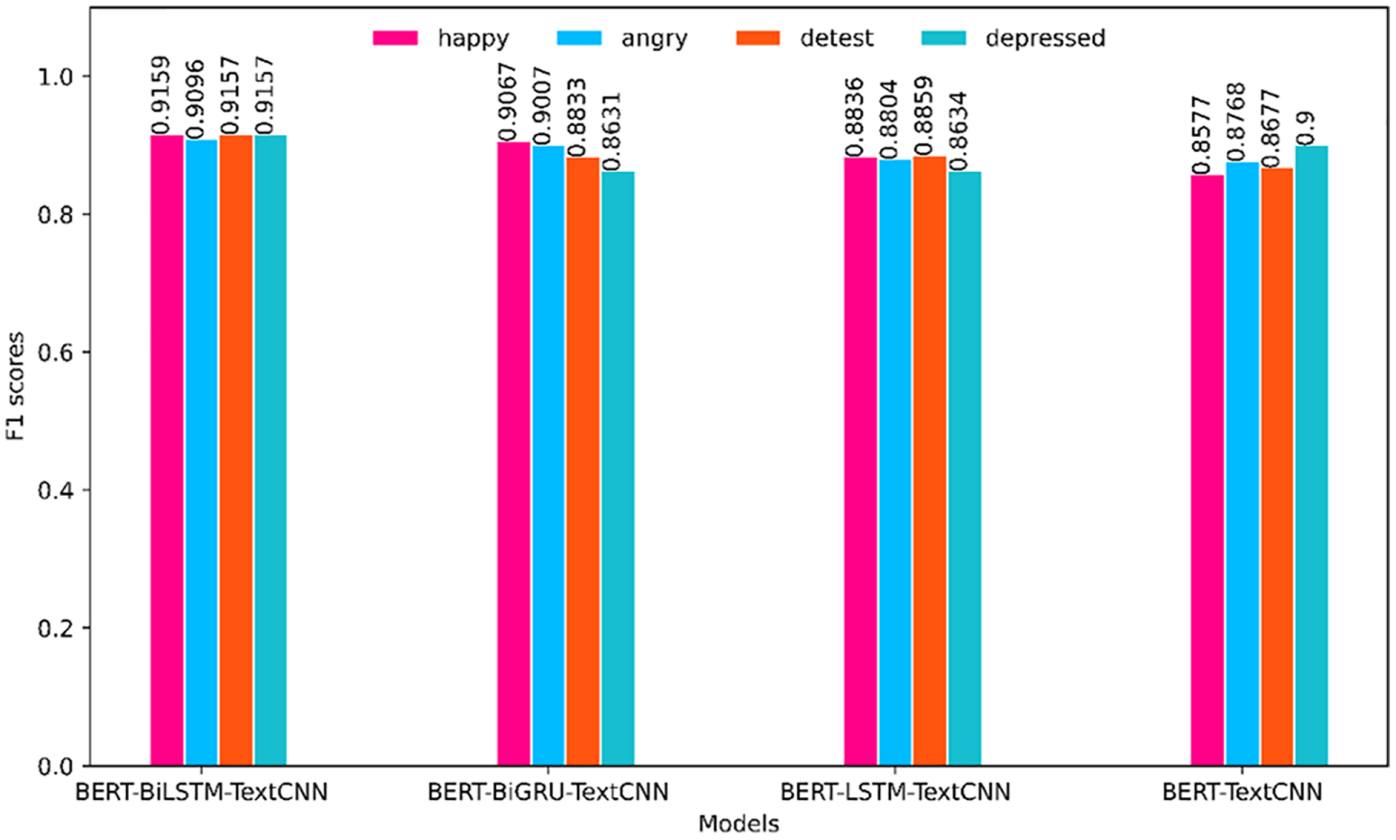
Comparison of F1 values of each category (based on BERT).

**Figure 5:**
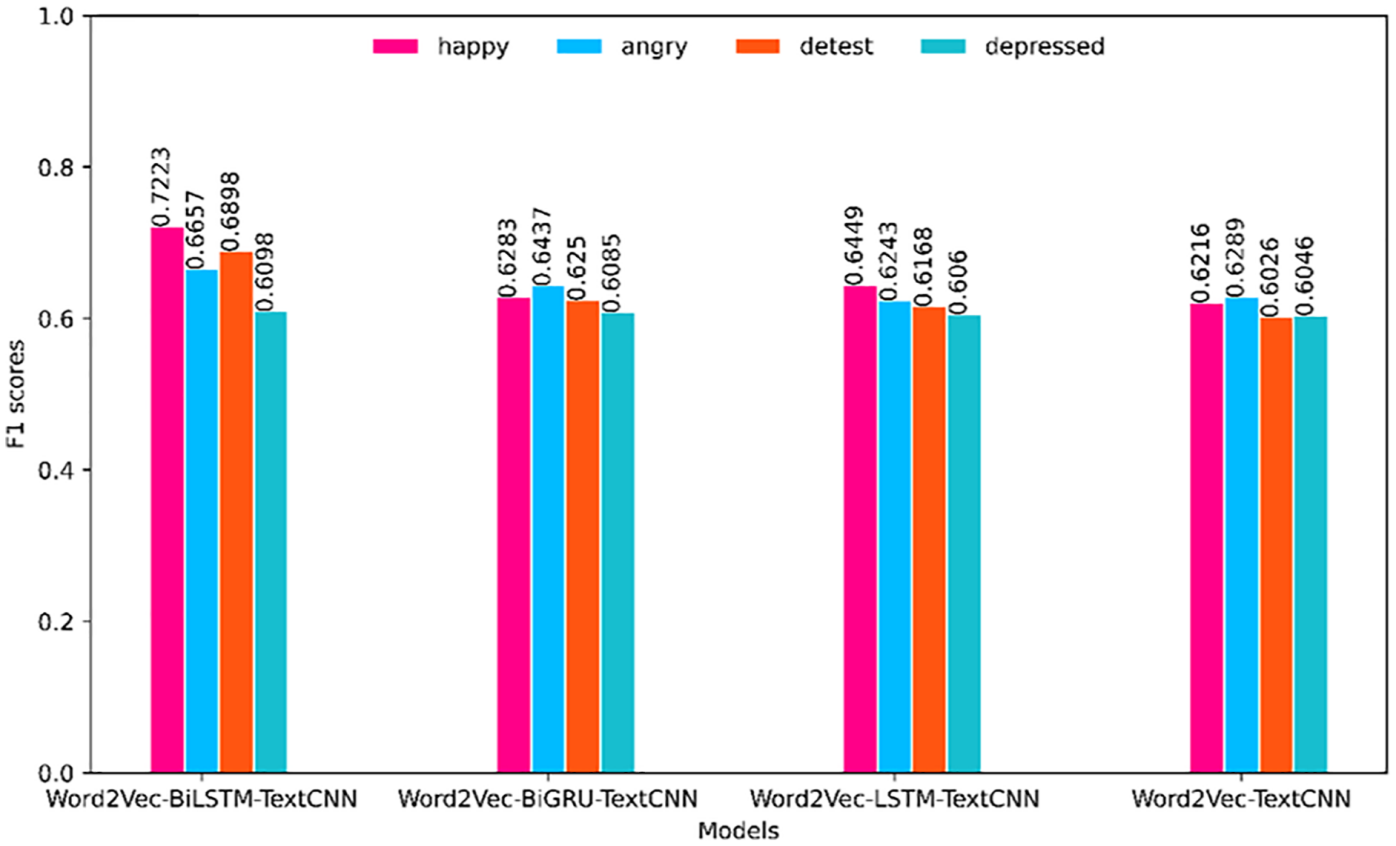
Comparison of F1 values of each category (based on Word2Vec).

To facilitate further comparison of the superiority of each model, this article divides the models into two groups for visual comparison in the horizontal direction according to the different embedding methods (BERT and Word2Vec). The comparison of the F1 value is shown in Figs. 4 and 5. The prediction effect of BERT-BiLSTM-TextCNN in each category is relatively stable, and the F1 value is generally high. The rest of the models all have different degrees of preference for certain types of sentiments, the prediction effect is less stable, and the prediction accuracy is lower than that of the target model.

**Figure 6:**
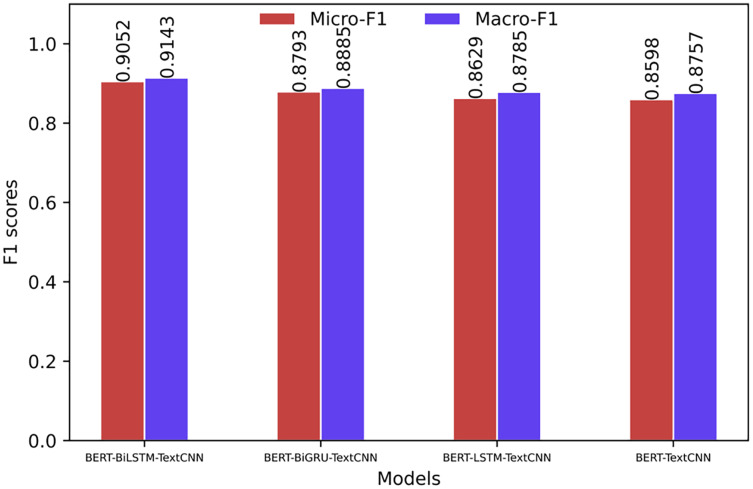
Overall evaluation of the model (based on BERT).

**Figure 7:**
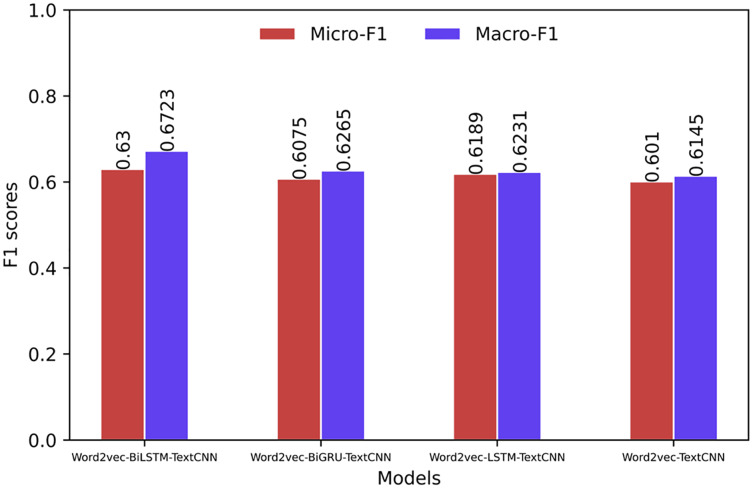
Overall evaluation of the model (based on Word2Vec).

The comparison of the Micro-F1 and Macro-F1 values showing the overall characteristics of the model is shown in Figs. 6 and 7. The Micro-F1 and Macro-F1 values of the target model are as high as 0.9052 and 0.9143, respectively, and the rest of the models are all lower than the target model.

To deeply analyze the effect of the model, five typical examples in the test set are selected for analysis, and the results are shown in Table 3. Label represents the actual emotion, Pre1 represents the prediction result of the target model BERT-BiLSTM-TextCNN, and Pre2 represents the prediction result of the benchmark model BERT-TextCNN. The category of the first comment is disgust, and the classification of the target model is correct, but the benchmark model is wrongly classified as joy. Compared with the target model, the benchmark model lacks the BiLSTM module and cannot extract contextual information well. The target model can compare A good point to identify the comment is in the second half of the sentence. The second sentence also involves the focus of the text. Because the target model fully extracts the context and comprehensively considers the meaning of the entire sentence, it completely judges that the emotional category belongs to “disgust”, while the benchmark model only focuses on this part of the sentence, leading to misclassification. The third sentence sentiment is relatively simple, and both models classify correctly. The emotion of the fourth sentence is more difficult to judge, with both anger and disgust, so the classification of the model has a certain degree of rationality. The fifth sentence is relatively difficult to classify, and it can be classified correctly by accurately identifying “vomiting”.

**Table 3:** Results analysis.

Comments	Label	Pre1	Pre2
You look very patriotic, very dedicated and very backbone, you never speak ill of others in the back and never frame others, forgive me for my just saying against my heart.	2	2	0
How could they call you a pig? This is outrageous! We can’t just call people what they look like!	2	2	1
He was so good at it that he broke another plate while doing the dishes. What a talented man!	1	1	1
You think you’re the sun, and everyone else must be around you. You know, there is only one earth in the universe, and it is probably burned by the arrogance of you.	1	2	2
If you ever learn to be sincere, I think the people around you will no longer vomit after you turn around.	2	2	2

Combining these classification examples, it can be found that the model is less effective for some meaningful text classifications, and there are also some misclassifications affected by the *corpus*, but overall, the problem of semantic inversion in comments has been solved to a certain extent. solve. Through further analysis, each module of the target model has played a crucial role: the TextCNN module can flexibly extract important emotional information in the text, and the effect of the benchmark model shows that when the comments are semantically reversed, only using TextCNN cannot accurately extract the sentiment information in the comments. We accurately extract sentiment information from reviews. When the text contains a variety of emotions, the BiLSTM module can accurately select keywords to obtain relatively accurate sentiment classification.

## Promotion Analysis

In this section, to further explore the generalization ability and stability of the BERT-BiLSTM-TextCNN model, we crawled micro-blog comments related to the Longping Yuan incident. The collection time was set from 21 May 2021, to 27 May 2021, and 240,000 comments related to Longping Yuan’s death were crawled. According to the theory of public opinion communication and micro-blog’s intelligent recommendation algorithm, the incident can be divided into three stages: (1) the outbreak peak: from 21 May 2021 to 22 May 2021; (2) the comprehensive spreading period: from 23 May 2021 to 24 May 2021; and (3) the control recovery period: from 25 May 2021 to 27 May 2021.

Through text cleaning, remove @ in the text, special characters such as emoticons, URLs, emails, and html codes, and special characters such as % in URLs, and convert traditional characters to simplified characters, etc., and get comments Corpora. Based on the above review corpora, the model is tested and compared with other models (Table 4).

**Table 4:** Evaluation results based on comment corpora.

Model	Micro F1	Macro F1
BERT-BiLSTM-TextCNN	0.9457	0.9503
BERT-BiGRU-TextCNN	0.9182	0.9071
BERT-LSTM-TextCNN	0.8836	0.8865
BERT-TextCNN	0.8471	0.8524
Word2Vec-BiLSTM-TextCNN	0.6836	0.6709
Word2Vec-BiGRU-TextCNN	0.6593	0.6684
Word2Vec-LSTM-TextCNN	0.6519	0.6476
Word2Vec-TextCNN	0.6411	0.6539

**Figure 8:**
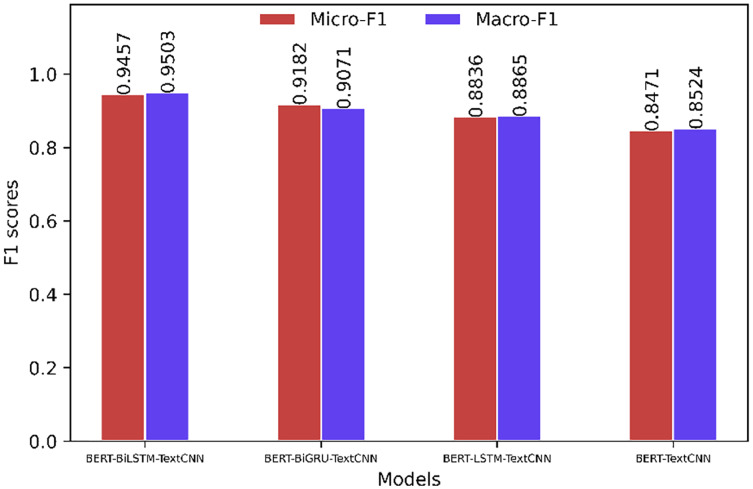
Model evaluation on the comment corpora (based on BERT).

**Figure 9:**
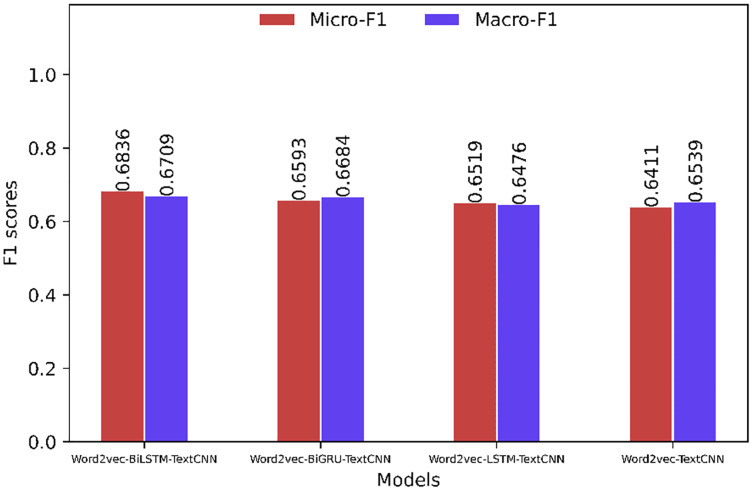
Model evaluation on the comment corpora (based on Word2Vec).

Figures 8 and 9 show that when the sentiment classification of the comment corpora is performed, the prediction accuracy is improved compared to the “simplyweibo_4_moods” data set. Through the analysis of the specific content of the corpora, we can see that most of the comments in the comment corpora have obvious emotional tendencies, and the content of the comments rarely has problems such as semantic inversion. Limited, but the content involved in the “simplyweibo_4_moods” data set is more extensive, so the model effects are quite different. However, from the existing evaluation indicators, the effect of the BERT-BiLSTM-TextCNN model is still the best. In addition, the effect gap between the BERT-BiLSTM-TextCNN model and the BERT-BiGRU-TextCNN model increases in the comment corpora because BiGRU has a great advantage in processing long text corpora, and most of the reviews are short. Therefore, the above experiments prove that BERT-BiLSTM-TextCNN has high effectiveness and accuracy in the task of reviewing sentiment classification on the network platform.

## Conclusion

Addressing the problems of missing information and partial information leakage in the word vector of the traditional language model and the problem that the existing sentiment analysis model cannot simultaneously and fully capture the long-distance semantic information and local semantic information. To address the problems of missing information and partial information leakage in the word vector of the traditional language model and the inability of existing sentiment analysis models to capture both long-distance semantic information and local semantic information simultaneously and adequately and to better solve the problem of prediction accuracy. Due to the problem of low prediction accuracy caused by colloquialism, open word range, and partial semantic reversal, a BERT-BiLSTM-TextCNN hybrid neural network model is proposed. The model first uses the pretrained BERT model for word vector representation, and then it acquires contextual information through the BiLSTM layer. The layer obtains the context information, enters the TextCNN layer to capture the local correlation, and finally performs the sentiment classification output through the softmax function. The experimental results show that, compared with seven models, such as BERT-BiGRU-TextCNN and BERT-LSTM-TextCNN, the BERT-BiLSTM-TextCNN model proposed in this article has the highest classification accuracy and is the most stable model. The experimental results show that (1) the hybrid model proposed in this article can better combine the advantages of BiLSTM and TextCNN, which can capture local correlation while retaining context information and has high accuracy and stability. (2) Compared with the suboptimal model BERT-BiGRU-TextCNN, the target model BERT-BiLSTM-TextCNN is improved by 2.75% and 4.32% in the evaluation of the indicators Micro-F1 and Macro-F1, respectively, which can accurately realize the multiclass classification task of emotions. (3) Each module of the target model plays a crucial role: the TextCNN module can flexibly extract important emotional information in the text, but when the text contains multiple emotions, it needs to cooperate with the BiLSTM module to accurately select keywords. Therefore, a relatively accurate sentiment classification can be obtained.

The model proposed in this article is not without flaws. First, the interpretability is not strong; it can only make predictions but not inferences. Second, when tuning the model, since there is no concept of feature importance in the TextCNN layer, it is difficult to adjust the specific features according to the training results. Later, a model can be found that can replace TextCNN and improve it.

## Supplemental Information

10.7717/peerj-cs.1005/supp-1 Supplemental Information 1 Data and code.

## Additional Information and Declarations

### Data Availability

The following information was supplied regarding data availability: The raw measurements are available in the Supplemental Files. The public data set is available at GitHub: https://github.com/SophonPlus/ChineseNlpCorpus/tree/master/datasets/simplifyweibo_4_moods.
